# Segmental zoster paresis of unilateral upper extremity: A case report and literature review

**DOI:** 10.1097/MD.0000000000020466

**Published:** 2020-07-10

**Authors:** Guan-Bo Chen, Sheng-Hui Tuan, I-Hsiu Liou, Hung-Ya Huang, Ya-Chun Hu, Shin-Yi Wu

**Affiliations:** a Department of Internal Medicine, Kaohsiung Armed Forces General Hospital; b Department of Rehabilitation Medicine, Cishan Hospital, Ministry of Health and Welfare; c Department of Physical Therapy, Shu-Zen Junior College of Medicine and Management; d Department of Physical Medicine and Rehabilitation, Kaohsiung Veterans General Hospital, Kaohsiung, Taiwan.

**Keywords:** case report, herpes zoster, nerve conduction study, rehabilitation, segmental zoster paresis

## Abstract

**Rationale:**

Segmental zoster paresis (SZP) is a relatively rare neurologic complication of herpes zoster (HZ), and is characterized by focal asymmetric motor weakness in the myotome that corresponds to skin lesions of the dermatome. The upper extremities are the second most commonly involved regions after the face, and predominantly involve proximal muscles. The pathogenesis of SZP remains unclear; however, most of the reports indicate that it is the inflammation because of the spread of the herpes virus.

**Patient concerns:**

A 72-year-old man without trauma history of the left shoulder joint developed weakness of the left proximal upper extremity 10 days after vesicular eruption of HZ.

**Diagnoses:**

His left shoulder girdle paresis was diagnosed with the upper truncus of the brachial plexus as a HZ complication according to a series of tests, including cervical magnetic resonance imaging (MRI), cerebral fluid analysis, sonography, and electrophysiological studies.

**Interventions:**

Acyclovir and prednisolone were administered during hospitalization to treat SZP. Meanwhile, analgesics and gabapentin were administered to control the patient's neuralgic pain. He also received inpatient (daily) and outpatient (3 times per week) physical therapy along with range of motion and strengthening exercises.

**Outcomes:**

Partial improvement of the strength of the left shoulder girdle, and no improvement of the left deltoid muscle was observed 2 months after the interventions.

**Lessons:**

This case emphasizes that HZ infections may be complicated by segmental paresis and they should be considered in the differential diagnosis of acute paresis in the upper limb. Awareness of this disorder is important because it avoids unnecessary invasive investigations and interventions, leading to suitable treatments with favorable prognosis.

## 1 Introduction

Herpes zoster (HZ) or shingles is a common disease followed by the varicella-zoster virus (VZV) infection. The symptoms include specific dermatomal vesicular skin lesions with burning pain. VZV is latent in the dorsal root ganglia and is reactivated during malfunctioning of the immune system. The commonly involved nerve segments are the thoracic, lumbar, and trigeminal; the incidence of typical HZ is approximately 4 to 4.5 per 1000 person years.^[1]^ Segmental zoster paresis (SZP) of limbs is the focal, asymmetric neurogenic weakness that may occur in an extremity affected by HZ.^[2]^ SZP is a relatively rare complication, whereby motor involvement can be observed in 0.5% to 5% patients with HZ.^[3,4]^ SZP typically occurs 2 to 3 weeks after the herpetic rash and affects the myotome that corresponds to the regions of rash distribution.^[5]^ In an electromyographic study, Mondelli et al found that SZP was 19% in 158 cases, and they concluded that the true prevalence of SZP may be underestimated because approximately 70% of the patients develop HZ in regions supplied by the ophthalmic branch of the trigeminal nerve, upper cervical (C2–C3) or thoracic nerve roots; therefore, any motor involvement may be difficult to detect in clinical practice.^[6]^ The existing SZP literature primarily includes case reports and the most commonly involved region is the face, accounting for approximately half the cases. The second most common region of SZP is reported to be the upper extremity with a predominant involvement of the proximal muscles (C5–C7).^[2,7]^

The pathogenesis of SZP remains unclear. Viral spread from the dorsal root ganglion to the anterior horn cells or anterior spinal nerve roots was suspected based on the association between the involved myotome and dermatome of the rash. The viral spread may cause inflammation that may cause a neurologic deficit, leading to hypervascularity in the perineural structures or actual disruption of the blood nerve barrier.^[7,8]^ However, these findings are still uncertain because the incidence of SZP is rare and the manifestation of both signs and electromyography (EMG) vary from patient to patient.^[4,9]^

The treatment of SZP is similar to that of HZ and primarily comprises antivirals and corticosteroids. The prognoses for patients with SZP are generally favorable after treatment.^[4,7]^ Proper management of a patient with SZP is undoubtedly based on a correct diagnosis. Given that this is still under-recognized by physicians, misdiagnoses or treatment delays may occur. Therefore, physicians must recognize and include SZP as a differential diagnosis for the acute onset of paresis. For this purpose, we report a case of segmental paresis of the upper extremity that is attributed to HZ to remind physicians of the disease and its rare motor complications.

## 2 Case report

Here we report a case study on a 72-year-old male patient who was consulted for left shoulder pain and weakness upon hospitalization to the Rehabilitation Department of Cishan Hospital. The patient provided informed consent for the publication of this report. We presented a detailed analysis of the SZP disease course and review of the literature based on the CARE guidelines. The patient complained of burning-like pain from his left shoulder to left forearm, followed by a vesicular eruption at the same location 10 days prior to admission. The diagnosis of HZ was made by a neurologist. An oral antiviral therapy with acyclovir was administered. Approximately 1 week later, the pain in the left elbow and forearm subsided. However, the patient was unable to elevate his left arm to the shoulder level, and the pain over the left shoulder persisted. Therefore, he visited our neurologic outpatient department where prednisolone (40 mg) was administered 2 times per day. The patient was admitted for further evaluation and management. His medical history included hypertension, coronary artery disease, and benign prostate hyperplasia. There was no history of trauma related to the left shoulder joint, or any systemic illnesses, including diabetes mellitus, cancer, or immunological disorder.

**Figure 1:**
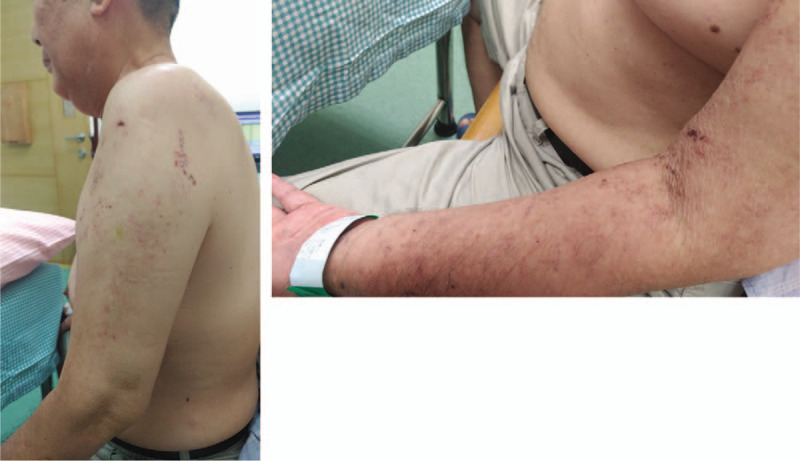
Skin lesions of tested patient with segmental zoster paresis. Physical examination of the patient revealed hyperpigmented, macular lesions from the left shoulder to the left forearm. Most of the skin lesions were over the left shoulder. The skin lesions began to heal after the Herpes zoster infection.

Physical examination of the patient revealed hyperpigmented macular lesions from the left shoulder to left forearm (Fig. 1) and most of the skin lesions were found on the left shoulder. Cervical spine motions were slightly painful with minimal limitations pertaining to lateral flexion. No radicular pain was provoked by the Spurling test. Left shoulder movements were painful and limited. The passive range of motion (ROM) of the left shoulder could attain the full range but in the presence of pain, whereas the active ROM of the left shoulder was severely limited according to the following conditions: flexion: 30°, extension: 30°, abduction: 15°; external rotation: 5°, internal rotation: 35°, and full adduction. Limited active flexion and abduction were partially compensated by scapulothoracic motion. The sulcus sign was negative in the left shoulder. There was no marked atrophy or fasciculation in the left shoulder muscles. ROM of the left elbow, wrist, and finger were normal and free of pain.

**Figure 2:**
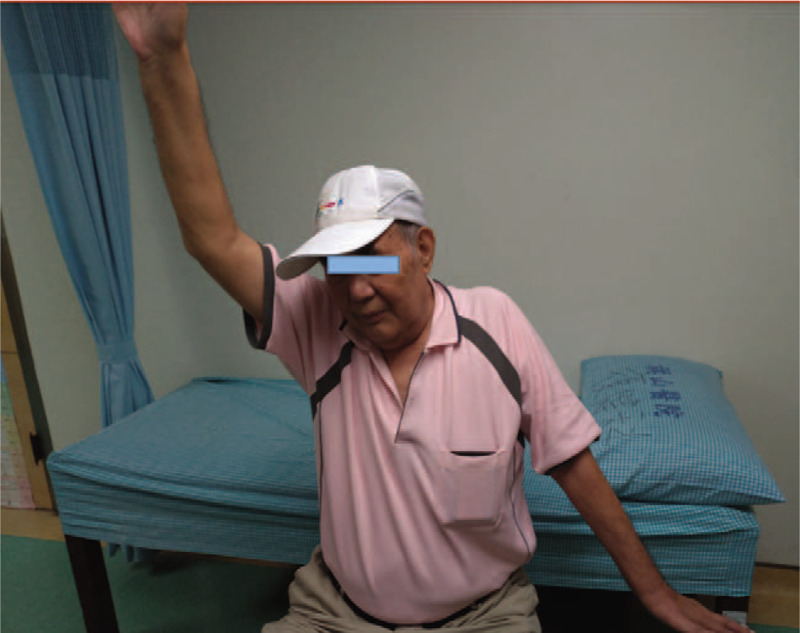
Weakness of left upper extremity of our patient with segmental zoster paresis. Compared with the sound site, the patient could barely elevate his left upper extremity when he performed an active test by himself.

Neurological examination revealed that the left biceps and brachioradialis reflexes were hypoactive and the triceps reflex was bilaterally normal. Marked weaknesses were found in the left deltoid, supraspinatus, subscapularis, and infraspinatus muscles [manual muscle testing (MMT) 2/5], whereas slight loss of muscle strength (MMT 4/5) was documented in the left biceps brachii muscles (Fig. 2). The muscle strengths of the left triceps, wrist extensors and flexors, and finger extensors and flexors were not compromised (MMT 5/5). In addition, hypoesthesia was found in all dermatomes in the range of C5 to C8.

Laboratory investigations revealed normal hemogram, erythrocyte sedimentation rate, C-reactive protein, and biochemical tests. Lumbar puncture was performed, and the cerebrospinal fluid was clear without any white blood cells or pleocytosis. Protein and glucose in the cerebrospinal fluid were within normal limits. Brain computed tomography revealed no peculiarities other than mild cerebral atrophy. There was mild spondylosis of the cervical spine with marginal spur formation and narrowing of the C5 and C6 disk space in the cervical spine radiograph. Further radiological investigation with MRI of the cervical spine revealed mild neural foramen stenosis at C4 and C5 to C6 and C7 without the evidence of nerve root compression. Left shoulder sonography revealed only spurring of the greater tuberosity without evidence of rotator cuff tendon injury. As the weakness in the left arm could not be explained using imaging studies or other investigations, an electrophysiological test was performed on the fourth day of admission.

A bilateral nerve conduction study was routinely performed for the median and ulnar nerves.^[10]^ The brachial plexus was stimulated 2.5 cm above the clavicle at the level of the 6^th^ cervical vertebra, at the triangular localization between the clavicle and posterolateral line of the sternocleidomastoid muscle. The motor response of the musculocutaneus nerve was recorded from the biceps muscle, and the axillary nerve response was recorded from the deltoid muscle using superficial electrodes.^[11]^ The amplitudes of the motor responses were decreased for the left median and left axillary nerves, while it was normal for the left musculocutaneus nerve compared with the nerve on the contralateral side. Sensory nerve conduction studies revealed relatively low-sensory nerve action potential amplitudes for the left median (recorded on the second digit) and left radial (record on the snuffbox) nerves with normal conduction velocities compared with the right side. No response was obtained from the lateral antebrachial cutaneous nerve. Normal motor and sensory responses were obtained from the peripheral nerves, which innervate the lower trunk (Table 1). Needle EMG showed spontaneous denervation activity and few motor unit potentials in the left deltoid, supraspinatus, and infraspinatus muscles. Needle evaluation of the left biceps brachii muscle showed only a mildly reduced recruitment pattern without spontaneous denervation activity. Normal motor unit potentials and recruitment patterns were observed in the left cervical paraspinal muscles, left rhomboid, and other muscles innervated by the lower trunk of the left brachial plexus.

**Table 1:**
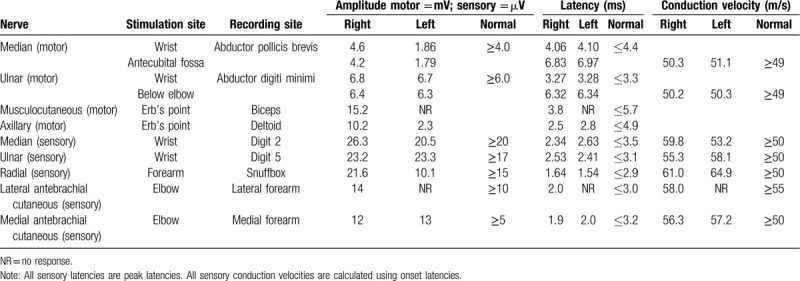
Data of nerve conduction studies of our patient with segmental zoster paresis.

The history, physical examination findings, biochemical, imaging, and electromyographic studies led to the diagnosis of SZP. Acyclovir and prednisolone were administered to treat SZP. Meanwhile, the patient was administered analgesics and gabapentin to control his neuralgic pain. A physical therapy program for daily exercise was initiated along with ROM and strengthening exercises. The patient was instructed to perform a home exercise program before hospital discharge. He confirmed during the examination that the pain intensity decreased 2 and a half weeks after his discharge from the outpatient clinic. The examination revealed increased shoulder ROM without improvement of muscle strength. The skin lesions had regressed but were still present. Mild atrophy of the left deltoid and supraspinatus muscles was detected. Outpatient physical therapy programs with ROM, strengthening exercises, and extra electrical stimulation in the left deltoid and supraspinatus muscles for 15 minutes and low-level laser therapy over left supraclavicular region for 10 minutes were conducted 3 times a week. The muscle strengths of the left biceps brachii (MMT 4/5 to full) and left supraspinatus and infraspinatus (MMT 2/5 to 3/5) muscles showed improvement; however, there was no change in the muscle strength of the left deltoid (MMT 2/5), and no progression of the atrophy was observed in the left deltoid and supraspinatus muscles at the follow-up visit 2 months later. The patient complained of low-intensity pain, and indicated a slight improvement in his left shoulder ROM.

## 3 Discussion

We reported a patient who presented with weakness in the left proximal upper extremity after HZ infection, and SZP diagnosis was ascertained after a series of laboratory and imaging examinations. SZP is reflected in a wide spectrum of clinical presentations. Based on the electrophysiological study by Cockerell et al, SZP represents a slow extension of viral inflammation from the sensory ganglion to the spinal cord, roots, plexus, and peripheral nerve.^[12]^ The reported estimate of segmental limb paresis with cutaneous zoster is approximately 3% to 5%.^[7]^ However, the true incidence of SZP may be underestimated because of the difficulties in clinical diagnosis of thoracic or upper cervical motor weakness, the pain often masking voluntary movements, and the weakness of SZP.

In a review of the literature by Sumihiro et al, SZP almost equally affects the upper and lower limbs.^[7]^ However, in a recent retrospective study of SZP using MRI and electrophysiological studies, Liu et al observed that SZP affected the upper limbs more often.^[4]^ There is a high incidence of HZ in individuals aged > 40 years, but the highest incidence is found in the age group of 60 to 70.^[1]^ Elderly patients with HZ develop motor complications more often than pediatric and young adult patients. However, when younger patients with VZV infections develop SZP, the cranial and truncal muscles are often affected.^[13]^ Immunocompromization, smoking, and diabetes are associated with neurological complications that follow VZV infection.^[14,15]^ Our patient was 72-year-old with a smoking history (smoked >1 pack per day for >30 years, but quit 10 years before the onset of this disease) and had no systemic disease other than hypertension and coronary artery disease.

Radiculopathy is a common form of HZ-related motor weakness.^[8,16,17]^ Motor weakness is the most frequent in segments C5 to C7 in the upper limbs and in segments L1 to L4 in the lower limbs of patients with SZP; the distribution of muscle weakness is common in proximal muscles of the limbs and limb-girdles.^[4,7]^ In a few cases, HZ resulted in partial or complete involvement of the brachial plexus. Eyigör et al reported a case of SZP in a 54-year-old male patient with partial involvement of the upper, middle, and inferior trunks of the brachial plexus.^[18]^ Rabia et al reported a 57-year-old male patient with SZP and involvement of the upper and middle trunks of the brachial plexus after VZV infection.^[19]^ Liu et al studied SZP in 8 Chinese patients and identified 2 cases with radiculopathy, 2 with plexopathy, 3 with radiculoplexopathy, and 1 with combined radiculopathy and mononeuropathy after electrophysiological testing.^[4]^ Our patient exhibited involvement of the upper trunk of the left brachial plexus after electrophysiological testing.

In cases where paresis follows an HZ infection, the diagnosis is established based on the history of weakness following painful skin lesions after exclusion of other causes of weakness and electrophysiological tests. Usually, the muscle weakness occurs days after occurrence of skin vesicles.^[19]^ However, the interval between the occurrence of the skin lesions and muscle weakness following a VZV infection vary. One case showed development of motor paresis 3 days before that of the skin lesions,^[20]^ whereas a few cases showed that patients exhibited weakness months after the rash.^[21,22]^ In this case, the patient showed weakness 10 days after vesicular eruption.

Given that the dorsal root ganglion is the site of VZV before HZ eruption, nerve conduction studies usually disclose reduced sensory nerve action potential amplitudes in the affected segments.^[6]^ Studies from Liu et al^[4]^ and Sachs et al^[23]^ proved that peripheral neuropathy caused by HZ may be primarily attributed to axonopathy. Therefore, needle EMG generally shows abnormal spontaneous activities, such as fibrillations and positive sharp waves, in clinically weakened muscles owing to SZP. However, the pathogenesis of SZP remains unclear. Postmortem observations demonstrated the degeneration of anterior spinal roots with lymphocytic infiltration of the posterior and anterior horns in patients with HZ.^[24]^ Pathologic studies observed demyelination, axon degeneration and lymphocyte infiltration in the affected nerves, dorsal root ganglions, and dorsal horns in patients with HZ.^[25,26]^ MRI studies revealed that some but not all the patients.^[4,27]^ with zoster paresis exhibited nerve enlargement and transverse relaxation (T2)-dependent signal hyperintensities in the dorsal horn, brachial plexus, or peripheral nerves. These suggested the hypervascularity or disruption of the blood–nerve barrier caused by viral-induced inflammation.^[8]^

Some case reports suggested that an antiviral treatment following an HZ infection could be effective in reducing the incidence of SZP and the severity of electrophysiological changes.^[6,19]^ Antiviral therapy may reduce peripheral sensory axonopathy and prevent viral spread.^[28]^ Moreover, given that there may be more extensive inflammation in patients who developed SZP than that shown in usual cutaneous zoster, corticosteroids were recommended for use immediately after the diagnosis of SZP.^[7,19,28]^ However, the efficacy of these treatments lack evidence rooted on controlled clinical trials. Pain relief, prevention of muscle atrophy and contractures, and the strengthening of weak muscles should be considered in the treatment of SZP. Although evidence to support rehabilitation on SZP is lacking currently, they are expected to play an important role in motor recovery.^[7]^ The prognosis for SZP is generally favorable with complete or partial recovery rates up to 75%.^[2]^ The recovery time can vary significantly; however, most of the presented cases varied between 1 and 2 years.^[2,29]^ The patient in this case was administered antiviral medication on occurrence of the skin vesicles. He was also given prednisolone because of the systemic weakness. Analgesics and gabapentin were used to control his neuralgic pain. Rehabilitation started as soon as his weakness developed. Improvement of muscle strength of most of the left proximal upper limb was detected 2 months after his referral despite the fact that there was no change in the strength of the left deltoid muscle at that time point. A long-term follow-up is expected to yield a more precise determination of the prognosis for this patient.

## 4 Conclusion

In conclusion, ZP secondary to HZ should be considered in the differential diagnosis of acute paresis in the upper limb. Awareness for this disorder is important given that it can avoid unnecessary invasive investigations and interventions, and could lead to suitable treatments with favorable prognoses. A good understanding of the neurologic complications secondary to HZ should be emphasized, while the rehabilitation is expected to play an important role in the motor recovery of patients with SZP.

## Author contributions

**Conceptualization:** Gaun-Bo Chen, Sheng-Hui Tuan, Ya-Chun Hu.

**Supervision:** I-Hsiu Liou.

**Writing – original draft:** Guan-Bo Chen, Hung-Ya Huang, Shin-Yi Wu.

**Writing – review & editing:** Sheng-Hui Tuan.
